# Artificial neural networks for quantitative online NMR spectroscopy

**DOI:** 10.1007/s00216-020-02687-5

**Published:** 2020-05-09

**Authors:** Simon Kern, Sascha Liehr, Lukas Wander, Martin Bornemann-Pfeiffer, Simon Müller, Michael Maiwald, Stefan Kowarik

**Affiliations:** 1 Bundesanstalt für Materialforschung und -prüfung (BAM), Richard-Willstätter-Str. 11, 12489 Berlin, Germany; 2 S-PACT GmbH, Burtscheider Str. 1, 52064 Aachen, Germany; 3 Bundesanstalt für Materialforschung und -prüfung (BAM), Unter den Eichen 44-46, 12203 Berlin, Germany; 4 Institute of Thermal Separation Processes, Hamburg University of Technology, Eißendorfer Str. 38, 21073 Hamburg, Germany; 5 Department of Physical Chemistry, University of Graz, Heinrichstr 28, 8010 Graz, Austria

**Keywords:** Online NMR spectroscopy, Real-time process monitoring, Artificial neural networks, Automation, Process industry

## Abstract

Industry 4.0 is all about interconnectivity, sensor-enhanced process control, and data-driven systems. Process analytical technology (PAT) such as online nuclear magnetic resonance (NMR) spectroscopy is gaining in importance, as it increasingly contributes to automation and digitalization in production. In many cases up to now, however, a classical evaluation of process data and their transformation into knowledge is not possible or not economical due to the insufficiently large datasets available. When developing an automated method applicable in process control, sometimes only the basic data of a limited number of batch tests from typical product and process development campaigns are available. However, these datasets are not large enough for training machine-supported procedures. In this work, to overcome this limitation, a new procedure was developed, which allows physically motivated multiplication of the available reference data in order to obtain a sufficiently large dataset for training machine learning algorithms. The underlying example chemical synthesis was measured and analyzed with both application-relevant low-field NMR and high-field NMR spectroscopy as reference method. Artificial neural networks (ANNs) have the potential to infer valuable process information already from relatively limited input data. However, in order to predict the concentration at complex conditions (many reactants and wide concentration ranges), larger ANNs and, therefore, a larger training dataset are required. We demonstrate that a moderately complex problem with four reactants can be addressed using ANNs in combination with the presented PAT method (low-field NMR) and with the proposed approach to generate meaningful training data.

**Electronic supplementary material:**

The online version of this article (10.1007/s00216-020-02687-5) contains supplementary material, which is available to authorized users.

**Figure:**
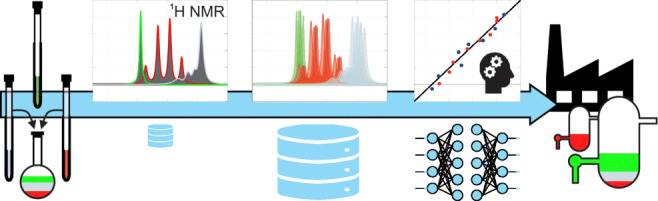
Graphical abstract

## Introduction

The process industry faces a growing demand for variable high-performance materials and specialized products with short setup times and a low resource consumption. This requires an increased use of digital technologies and a change of mind as this poses a major challenge to the industry and its suppliers. The measurement of specific process data by means of complex process analytical technology (PAT) can be quite complicated and expensive. Since the data pretreatment and evaluation in some cases has to be performed by people with a high level of competence, this leads to high costs which is why some process analytical procedures are often scarcely used.

Quantitative nuclear magnetic resonance (NMR) spectroscopy is today one of the most important instrumental analytical methods in the natural sciences and medicine, because of its ability to determine clear substantial and structural information nondestructively. Due to its direct quantitative nature, it is particularly important for the chemical, biochemical, and pharmaceutical industries, e.g., for purity analysis of organic compounds [1]. Apart from this, NMR spectroscopy can also serve as an online method for reaction and process monitoring [2–4] of fluids and technical mixtures (e.g., for process development) because it provides quantitative reference data that can be transferred to analytical methods operated in parallel. A recent application example suggests that NMR spectroscopy has significant potential to enter the production environments in process industry [5], making it a useful extension to the toolbox of PAT methods like optical process spectroscopy [6]. Further, NMR spectroscopy can be applied in a wide temperature and pressure range. The high linearity between absolute signal area and concentration makes it an absolute analytical comparison method to determine the concentrations of major and secondary components without altering the sample. Peak areas in the spectrum can be used directly for quantification, without the need for calibration—assuming complete spin magnetization is achieved. This is an important prerequisite for robust data evaluation strategies within a control concept and reduces the need for extensive maintenance of the evaluation model over the time of operation.

### Challenges

The use of low-field NMR spectrometers with permanent magnets is favorable for process monitoring tasks, due to lower space and maintenance requirements compared to high-field NMR spectrometers using helium-cooled superconducting magnets. Technical samples lack deuterated solvents, and usually multiple analytes and the solvent are present. This often results in complex and overlapping spectra which prevent the evaluation through direct integration of peaks to quantify chemical species in the mixture. Despite these overlaps, the NMR signals behave extremely linear and independent of the surrounding matrix, which is an excellent basis for research into data evaluation methods.

### Multivariate methods for quantitative analysis of spectra

There are several established methods to overcome these difficulties, like line fitting [7], indirect hard modeling (IHM) [5, 8, 9], model-based methods in time domain [10], and regression methods like partial least squares regression (PLS-R) [11, 12]. Whereas the model-based approaches require careful parameterization but less data to set up, data-driven methods such as PLS-R and artificial neural networks require a large amount of training data along with reference/meta-data.

Typically for PAT applications, the calibration and validation data need to be collected from time and cost-intensive lab experiments and the quality of multivariate models strongly depends on sampling, sample preparation, and the uncertainty of the reference analytical method. In order to map the diversity and variance of states in the process, this calibration should move along a design of experiments. Such modeling is typically very costly and can be even more expensive than the cost of the method itself. Furthermore, a transfer of the data from the process laboratory or R&D into real processes is often not possible because laboratory data does not include the disturbing factors occurring in the real process as these cannot be accounted for well enough in the laboratory. Later, the model must be constantly updated with real samples if, for example, the raw materials vary. In many cases, chemometric models can only represent what has been implemented during the calibration process, in addition to the considerable costs for the creation of such models, which have a strongly limiting influence on the use of PAT. Often, models fail when additional, untrained conditions occur, e.g., additional substances within the process and blocked windows, or when device components such as optical windows, optical fibers, light sources, or detectors wear out or age, to name an example of optical spectroscopy.

The ideal solution would be to have a measurement system that, based on its analytical methods and the appropriate calibration models, can provide the operator with more information than current models can. Such a “smart sensor” would be able to deliver valuable information on additionally occurring substances or unknown process conditions.

### Artificial neural networks

Machine learning is currently experiencing a huge technical and social interest, because machine-supported processes can take over complex tasks by learning from examples. Artificial neural network (ANN) algorithms are a subgroup of machine learning algorithms. They are particularly useful for problems where it is difficult to provide a simple model-based solution or data must be processed quickly due to either the large amounts of data or a real-time constraint [13]. The application of ANNs for quantitative [14–16] and qualitative [17] spectroscopy as well as chromatographic data [18] has already been described in the literature. In these studies, ANNs can help to improve accuracy and speed up the analysis of spectra but, using interpretable machine learning techniques, can also help to visualize important features in spectra [19]. ANNs are especially useful when linear models such as PLS-R fail due to a high level of complexity within the spectra.

ANNs are based on a collection of nodes or units that are typically arranged in layers between an input layer and the output of the ANN. The information between different layers of an ANN can be passed and processed in various ways, involving mathematical linear operations and typically nonlinear activation functions between nodes or layers. The *strength* (or weights) of the connections between the individual nodes between layers is typically adjusted, or *learned*, during the training phase by means of a backpropagation algorithm with the aim of minimizing a loss function. That means that the ANN is optimized to predict a minimal deviation of the ANN’s output compared to the ground truth of the training examples. Using input data, here NMR spectra, that are fed to the input layer of the trained model, it will then predict output results, in our case component areas and therefore concentrations. The generalization capability and accuracy of the models’ predictions very much depend on the quality and quantity of the training data as well as the training labels.

In this work, we have trained and tested two different ANN architectures: a simple multilayer perceptron (MLP) model, where all nodes between subsequent layers are directly connected, as well as convolutional neural networks (CNNs). CNNs perform particularity well on multidimensional data with spatial dependencies [13], such as image processing tasks and object recognition, but can also be applied to our 1D spectral data [17]. CNNs typically contain the following components: convolutional layer, application of a nonlinear activation function, and a subsampling layer. Each convolution layer extracts features from the incoming data (such as an image or spectrum) using a set of adaptive filters, called kernels. The convolution calculation is performed as a sliding dot product between the filter kernels and the input data. At the last stage of a CNN, a classifier or regression layer is used to extract the requested information.

Both the MLPs and CNNs need ample amounts of training data to make accurate predictions, and in some examples, the complexity of determining the ANN weights was compensated by increasing the amount of measured reference data. Bishop [13], Liu et al. [17], and Kästner et al. [20] described data augmentation procedures for spectral data based on linear combinations of measured spectra to increase the diversity of the data available for training ANNs.

The presented work shows a novel calibration concept for spectroscopic sensors using machine learning, which needs less calibration data than usual approaches. We hope that this simplifies the integration of smart sensors into the digital infrastructure. This supports not only real-time release and continuous manufacturing but also classic batch chemistry for fine chemicals. To overcome the issue of the large amount of training data needed for ANNs and the development of multivariate models, in this publication, simulated variants of the measured data (i.e., synthetic NMR spectra) are employed to avoid overfitting. This data augmentation procedure for the generation of synthetic but physically based NMR spectra enables the development of data-driven models such as neural nets because the initial training dataset can be sized and distributed along various prediction variables arbitrarily.

As an exemplary system, a lithiation reaction was monitored in a continuous flow mode in lab scale with a low-field NMR spectrometer (^1^H, 43 MHz). For validation purposes, a high-field NMR spectrometer (^1^H, 500 MHz) served as reference. To have enough data to train the ANNs towards the prediction of the reactants’ concentrations, different possibilities are available. Two approaches for the generation of synthetic ANN training data are proposed: (i) training data generated from combinations of measured pure component spectra and (ii) training data based on a spectral model, which incorporates nonlinear effects such as peak shifts and peak broadening. Within the following work, *X*_*i*_ refers to the “pure component spectral dataset” and *X*_*ii*_ refers to the “spectral model dataset.” The concentration data based on experimental low-field NMR spectra and reference values from the high-field NMR results are used to validate the proposed methods.

## Materials and methods

### Experimental methods

#### Chemical reaction

**Fig. 1:**
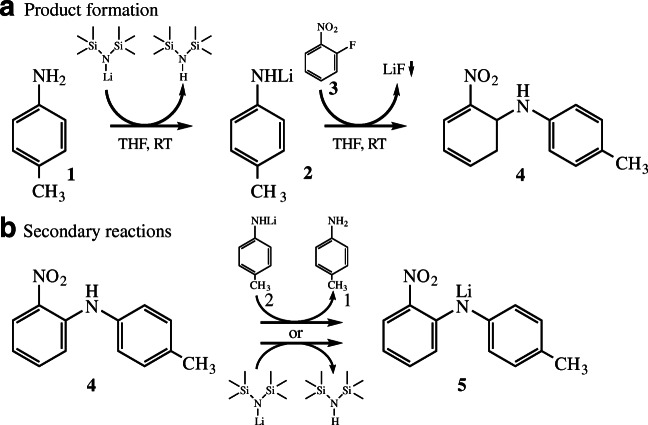
Reaction steps for coupling of *p*-toluidine (**1**) and 1-fluoro-2-nitrobenzene (**3**) induced by lithium bis(trimethylsilyl)amide and lithiated *p*-toluidine (**2**) as intermediate to nitro-4′-methyldiphenylamine (**4**). THF, tetrahydrofuran; RT, room temperature. **a** Product formation. **b** Secondary reactions

The synthesis of nitro-4′-methyldiphenylamine (MNDPA) by aromatic substitution of *p*-toluidine and 1-fluoro-2-nitrobenzene (*o*-FNB) is a relevant example for the pharmaceutical industry [5, 21]. *p*-Toluidine was activated by a proton exchange with the organolithium compound lithium bis(trimethylsilyl)amide (Li-HMDS). Figure 1 shows a scheme of the complete reaction network. After addition of Li-HMDS to the reagents (**1** and **3**), the proton exchange takes place between the primary amine (**1**) and Li-HMDS. In the second step, the product MNDPA (**4**) is formed by a substitution reaction of the produced lithiated toluidine (**2**) and *o*-FNB (**3**). LiF precipitates as a solid in the reaction solution due to its low solubility in tetrahydrofuran (THF) [22].

Subsequent reactions (Fig. 1b) are proton exchange reactions analogous to the first reaction step between the amine group of MNDPA (**4**) and a lithium compound (**2** or Li-HMDS), where the equilibrium of the respective reaction is completely on the product side (Li-MNDPA, **5**). This means that for the complete conversion of reagents, at least a twofold excess of Li-HMDS is required due to the abovementioned unavoidable secondary reactions.

#### Chemicals

The chemicals *p*-toluidine (Sigma-Aldrich, 99.6%), 1-fluoro-2-nitrobenzene (Sigma-Aldrich, 99%), THF (Chemsolute, > 99.9%), and lithium bis(trimethylsilyl)amide (Sigma-Aldrich, 1 mol L^−1^ in THF) were used without prior purification.

#### Experimental setup

**Fig. 2:**
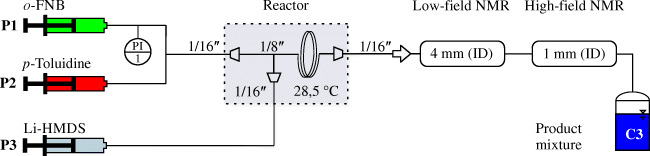
Experimental setup for monitoring of the continuous lithiation reaction with low-field and high-field NMR spectroscopy. The reaction (see Fig. 1) was performed continuously in a temperature-controlled 1/8-in. tubular reactor with syringe piston pumps (P1–P3)

To evaluate the robustness of various data analysis approaches for online spectra, a continuous reaction setup was implemented (Fig. 2). The reactants *p*-toluidine (0.83 mol L^−1^ in THF) and *o*-FNB (0.63 mol L^−1^ in THF) were dosed by a syringe pump (P1 and P2, Gemini 88; KD Scientific, Holliston, USA), premixed in a T piece, and subsequently mixed with 1 mol L^−1^ Li-HMDS (P3, Nemesys high-pressure syringe pump; Cetoni, Korbussen, Germany; with modified sealings). For stoichiometric conversion, the ratio of *p*-toluidine, *o*-FNB, and Li-HMDS was set to 1:1:2 which resulted in flow rates of 0.193 mL min^−1^, 0.254 mL min^−1^, and 0.353 mL min^−1^, respectively. After screening the edges of the reaction conditions (0 mL min^−1^ and 0.8 mL min^−1^ for each reactant), the flow rates were modulated with 90–150% of the stoichiometric flow rates. The overall flow rate for the tubular reactor was fixed to 0.8 mL min^−1^.

Based on step tracer experiments, the delay time between the change of a flow rate and the signal change of each spectrometer was set to 3.3 min and 8.8 min for low-field and high-field spectrometers, respectively. The flow cells consisted of a glass cell with 4 mm inner diameter (low-field NMR) and a 1/16-in. PFA tube (ID = 1 mm, high-field NMR).

#### Online NMR spectroscopy and basic data preparation

High-field NMR spectra (*S*_hf_) were acquired as reference spectra using a 500-MHz NMR spectrometer (Varian) with a medium-pressure broad band flow probe (^1^H/^15^N–^31^P). Online proton spectra during reaction monitoring were acquired with two scans, 45° pulses, 5-s acquisition time, and 15-s relaxation delay. The low-field NMR instrument (Spinsolve Proton; Magritek, Aachen, Germany) operating at 43.3 MHz proton frequency is equipped with a 5-mm bore for standard NMR tubes at a magnet temperature of 28.5 °C. Online proton spectra were acquired with single scans, a 90° pulse, 6.5-s acquisition time, and 15-s repetition time.

The acquired proton spectra were processed in MATLAB (R2019a). Apodization by exponential multiplication with a line broadening factor of 0.5 Hz was conducted. After Fourier transformation, spectra were immediately processed by automated methods, implemented in MATLAB. These are baseline correction [23], phasing [24], and spectral alignment to a THF spectrum using the icoshift algorithm [25]. Due to signal overlap in both high-field and low-field NMR spectra, quantification of reactants was performed using indirect hard modeling [26] as described in Kern et al. [8].

### Experimental reference data

A major advantage of NMR spectroscopy as an analytical tool is that the signal intensity in the spectrum is directly proportional to the number of nuclei responsible for this resonance. Peak areas can be converted into corresponding SI units such as the amount of substance concentrations or mass concentration by a simple linear conversion function. For simplicity, consequently, component areas were used within all calculations.

**Fig. 3:**
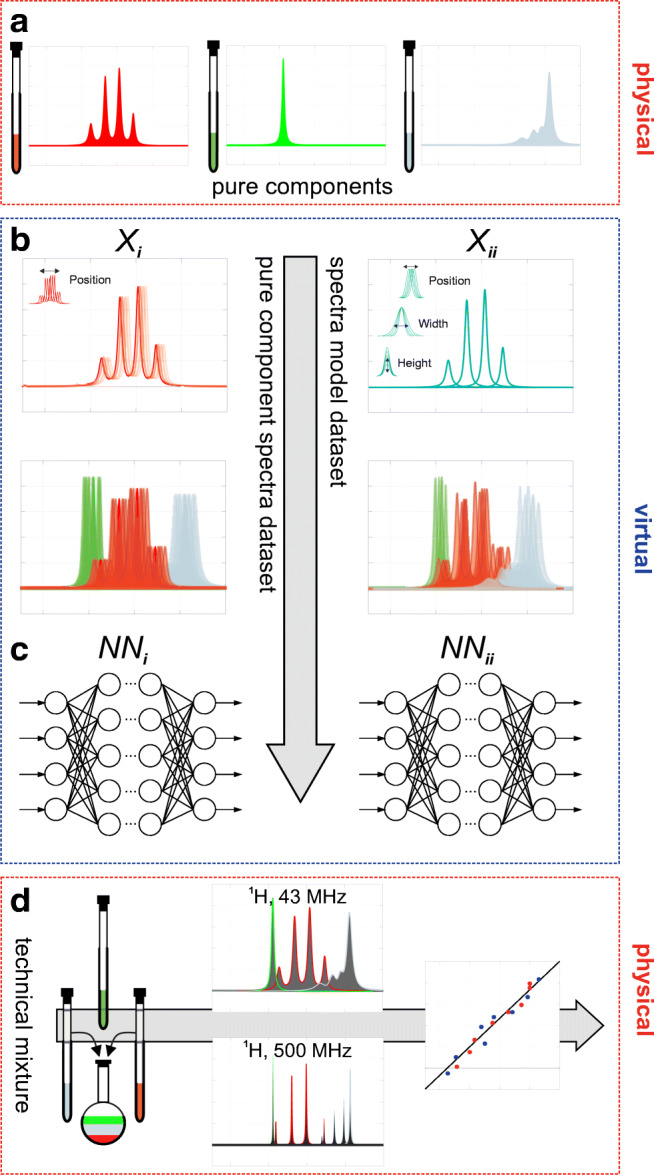
The workflow is divided in 4 steps (**a**–**d**). **a** Acquisition of pure component spectra for each species. **b** Calculation of 300,000 synthetic training spectra based on measured pure component spectra (*X*_*i*_) and spectral models describing these spectra through peak functions (*X*_*ii*_). **c** Setup and training of two independent neuronal networks (NN_*i*/*ii*_) with NMR spectrum as input and component areas as output. **d** Performance comparison of both ANN models and IHM compared to high-field NMR results

**Fig. 4:**
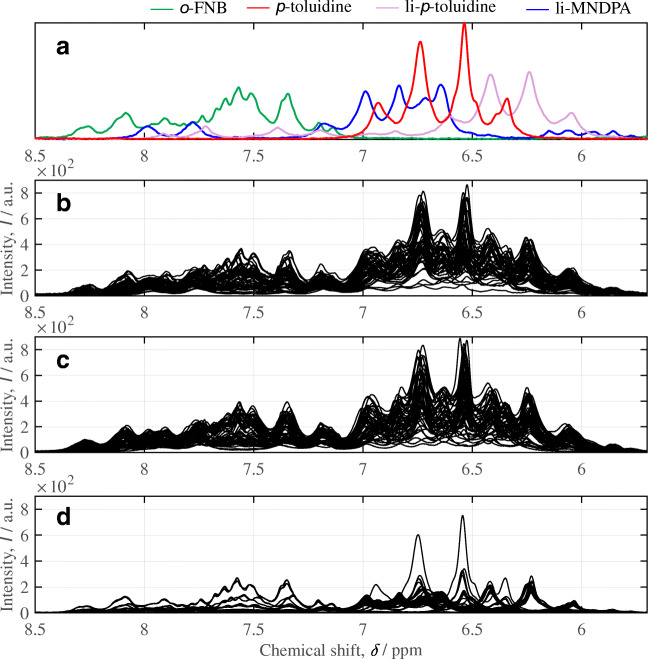
Low-field NMR spectra of continuous synthesis of nitro-4′-methyldiphenylamine (MNDPA). ^1^H spectra (43 MHz) were recorded as single scans. **a** Experimental pure component spectra. **b** 50 superimposed synthetic mixture spectra which were randomly generated based on the measured pure components (**a**). **c** 50 superimposed synthetic mixture spectra which were randomly generated based on the pure component models. **d** 150 experimental NMR spectra during MNDPA synthesis which were used for testing purposes

Reactants involved in the investigated aromatic substitution reaction show several protons with similar chemical shifts in the aromatic spectral range within the ^1^H NMR spectrum (see Figs. 3 and 4a). However, further aliphatic NMR signals from the solvent and the organolithium compound in low-ppm region (5.5 to − 1 ppm) were not taken into consideration. The spectral range which was considered for quantitative evaluation (5.6 to 9 ppm) showed much more complex signals in the low-field NMR spectrum (^1^H, 43 MHz) due to the increased line widths in comparison to high-field NMR spectra. In addition, due to the low field strength, higher-order spectra occur; i.e., the distance of the chemical shifts (*δ*_*i*_) within a spin system falls in the range of the coupling constants, so that the exact *δ*_*i*_ can usually no longer be interpreted visually from the peaks. Consequently, the respective area of each pure component in the low-field NMR spectra cannot be obtained directly from numerical integration of spectral ranges and has to be determined using multivariate methods or, as newly proposed in this work, using artificial neural networks. To overcome the issue of lacking training data, a data augmentation procedure is proposed and is based on simulated variants of the measured pure component spectra (hereafter called synthetic NMR spectra). The systematic approach used in this work is presented in Fig. 3 and henceforth described in detail.

#### Further data filtering and refinement

We validate the three different data evaluation models, which use baseline-corrected, phased, and aligned low-field NMR spectra with high-field NMR data. We consider only measurements during steady-state phases of the continuous reaction setup to prevent reference concentrations from being affected by concentration gradients moving through both NMR spectrometers (Fig. 2) while acquiring the spectra. In a first step, for each timestamp of low-field NMR spectra, a corresponding high-field NMR result was assigned by the nearest-neighbor interpolation, since both spectrometers did not acquire spectra at the same time. For each analyte concentration (derived from IHM), a moving linear fit was calculated from 11 consecutive online NMR concentration values. Linear fit results with both a slope below 0.1 mol h^−1^ and a standard deviation below 0.01 mol L^−1^ among the data points were classified as steady states. Additionally, outliers which showed greater deviations than 0.04 mol L^−1^ between high-field NMR and low-field NMR (from IHM) were removed.

The remaining dataset consisted of about 1700 spectra and reference values with a comparably high bias within the concentration ranges. The Kennard–Stone algorithm [27] was used to select 300 samples with a uniform coverage from the remaining dataset. The final experimental reference dataset consisted of the measured high-field NMR results (*A*_hf_) and low-field NMR spectra (*S*_lf_).

### Simulation of synthetic training spectra

The experimental mixture spectra from the continuous MNDPA synthesis (Fig. 4d) were mainly obtained for concentration ranges around the optimal stoichiometric conditions by systematically varying the pump rates in this range. Especially in industrial applications, it is of great importance to minimize the concentration of the remaining reagents in the product stream. Consequently, for calibration purposes, it is challenging to validate automated data analysis methods if prediction parameters are nonuniformly distributed within the training data. Therefore, we propose two possible methods for the generation of synthetic training data, which are uniformly distributed for all prediction parameters (Fig. 4b, c).

Each NMR spectrum of chemical mixtures can be considered as a linear combination of pure component spectra, due to the strictly linear correlation of signal area and concentration within the NMR method. However, online NMR experiments in continuous flow are usually subject to a large number of distortions in the NMR spectrum caused by inhomogeneities in the magnetic field such as temperature fluctuations of the magnet or solid particles in the sample. These nonlinear effects result in peak shape distortions (peak widening or asymmetric peaks) or peak shifts, which must be considered during the generation of synthetic mixture spectra. For all calculations within this section, the standard uniform pseudorandom number generator in MATLAB was used.

#### Training data based on combinations of measured pure component spectra

For the generation of synthetic mixture spectra based on a linear combination of measured pure component spectra, the parameters line broadening factor, component shift, and component area were uniformly distributed by random numbers in the specified range (Table 1). For every generated mixture spectrum, these parameters were generated for each of the four pure component spectra: *o*-FNB, *p*-toluidine, Li-*p*-toluidine, and Li-MNDPA. Subsequently, all altered pure component spectra are summarized to one mixture spectrum with known component areas. The component area was calculated by numerical integration of each altered pure component spectrum.

**Table 1:** Parameter ranges for the generation of mixture spectra based on pure component spectra (*X*_*i*_) based on experience (prior knowledge)

Component area (*A*_lf,synth_ / a.u.)	0–180
Line broadening factor / Hz	0.2, 0.5, 0.8, 1.0, 1.5
Component shift / ppm	0–± 0.015

By introducing alterations in each pure component spectrum, the distortions through abovementioned nonlinear effects are intended to mimic real process variations. In order to adjust the component area, each pure component spectrum was scaled by multiplication with a scalar.

The direct use of measured pure component spectra comprises two major limitations compared to a model-based approach. First, noise obtained with the measurement is scaled equally while altering the component area. Second, shifts of single peaks within a pure component spectrum due to mixing effects cannot be considered. Both limitations can be overcome using a spectral model, e.g., as published by Alsmeyer and Marquardt [28] and Kriesten et al. [9]. These are based on parametric models of the pure component spectra that are made flexible enough to describe typical variations in mixture spectra. Hereby, each pure component is modeled by a sum of peak functions (pseudo-Voigt functions).

#### Training data based on a spectral model

The model equations for the spectral model were adopted from Kriesten et al. [9] and extended with NMR-related parameters in order to allow more variance within the synthetic spectra. Pure component models of each reactant were generated in PEAXACT 4 by fitting pseudo-Voigt functions (*V*) to the respective measured pure component spectrum (see Electronic Supplementary Material (ESM) Fig. S1). Resulting peak parameters half width (*γ*), maximum (*α*), position (*ω*), and Gauss–Lorentz weight (*β*) were imported to MATLAB.

In order to group similar behaving signals in the pure component spectrum, the individual peak functions were divided into groups. Peak functions sharing the same group number are affected by one same group shift parameter (***θ***_GS,*k*_). The group number was automatically assigned to the peak functions. Peak functions whose position (*ω*) deviates by more than Δ*ν* = 0.12 ppm from the position of another peak were assigned to a new group. The value of Δ*ν* was determined from preliminary experiments. The peak broadening parameter (*θ*_PB,*k*_) was introduced to account for any broadening effects which apply to all peaks in the pure component. *θ*_PB_ is multiplied by all widths (*γ*) in the pure component model. The spectral mixture model (*χ*) can be formulated analogous to Kriesten et al. [9] by including the group shift parameter (***θ***_GS,*k*_) and the peak broadening parameter (*θ*_PB,*k*_).

1 $$ \chi \left(\nu, \boldsymbol{w},\boldsymbol{\theta} \right)={\sum}_{k=1}^K{w}_k{S}_k\left(\nu, {\boldsymbol{\theta}}_{\mathrm{P},k},{\theta}_{\mathrm{S},k},{\theta}_{\mathrm{GS},k},{\theta}_{\mathrm{P}\mathrm{B},k}\right), $$

where the vector ***θ*** contains all model parameters, i.e., the peak parameters of the pure component models (***θ***_P_ = (***θ***^T^_P,1_,…, ***θ***^T^_P,*K*_)^T^) and the component shift parameters (***θ***_S_ = (*θ*^T^_S,1_,…, *θ*^T^_S,*k*_)^T^). The spectral axis in ppm is represented by *ν*, the component weights by ***w***, and each *k*th component model by *S*_*k*_.

In order to generate the training data based on the spectral model (*χ*), the following model parameters have been varied within their ranges (Table 2) for calculation by the model: peak position (*ω*), component shift (*θ*_S,*k*_), group shift (*θ*_GS,*k*_), peak broadening parameters (***θ***_PB_), and weights (***w***). Additionally, signal noise from a measured and baseline corrected spectrum was added to the mixture model. Each data point from this spectrum (only baseline regions) was randomly selected and scaled with a noise factor.

**Table 2:** Parameter ranges for the generation of mixture spectra based on the applied spectral model (dataset *X*_*ii*_) based on experience (prior knowledge)

Peak position, *ω* / ppm	0–± 0.005
Component shift, *θ*_S,*k*_ / ppm	0–± 0.015
Group shift, *θ*_GS,*k*_ / ppm	0–± 0.01
Peak broadening parameter, *θ*_PB,*k*_ / %	0–± 15
Component area, *A*_lf,synth_ / a.u.	0–180
Noise factor / %	0–± 15

### Artificial neural networks

The first subsection describes the general training and validation procedure of the ANNs. Furthermore, it is specified how the training, validation, and test data were used. The ANN architecture and parameters are summarized in the second subsection. The NN predictions and a corresponding performance analysis are presented in the “Results and discussion” section.

#### Training approach

The training of the ANNs is conducted with the generated synthetic datasets: *X*_*i*_ (pure component spectral dataset) and *X*_*ii*_ (spectral model dataset). *X*_*i*/*ii*_ is randomly split into training datasets (*X*_*i*/*ii*,train_) and validation datasets (*X*_*i*/*ii*,val_) by a ratio of 95:5. During training, the model performance is evaluated on synthetic training data. Three hundred thousand synthesized spectra *X*_*i*/*ii*_ are used during training, respectively. The training labels are the respective areas of each underlying pure component spectra (*A*_lf,synth_). The ANN predictions of the actual component areas from low-field NMR measurement data (*S*_lf_) are labeled *A*_lf,ANN_.

The measured high-field NMR results (*A*_hf_) are used as ground-truth labels as they exhibit much higher accuracy than what can be obtained from low-field NMR spectra. *A*_hf_ is used as reference to calculate the ANN predictions’ relative error with the measured low-field NMR spectra (*S*_lf_) as model input. *A*_hf_ is also used as reference for a conventional IHM analysis [5, 8] (our reference method) from the same *S*_lf_ input. The deviations of the prediction values (*A*_lf,ANN_) and *A*_lf,IHM_ from the precise high-field NMR results (*A*_hf_) are quantified as mean squared error MSE (*A*_hf_, *A*_lf,ANN_) and MSE (*A*_hf_, *A*_lf,IHM_) throughout this work.

**Fig. 5:**
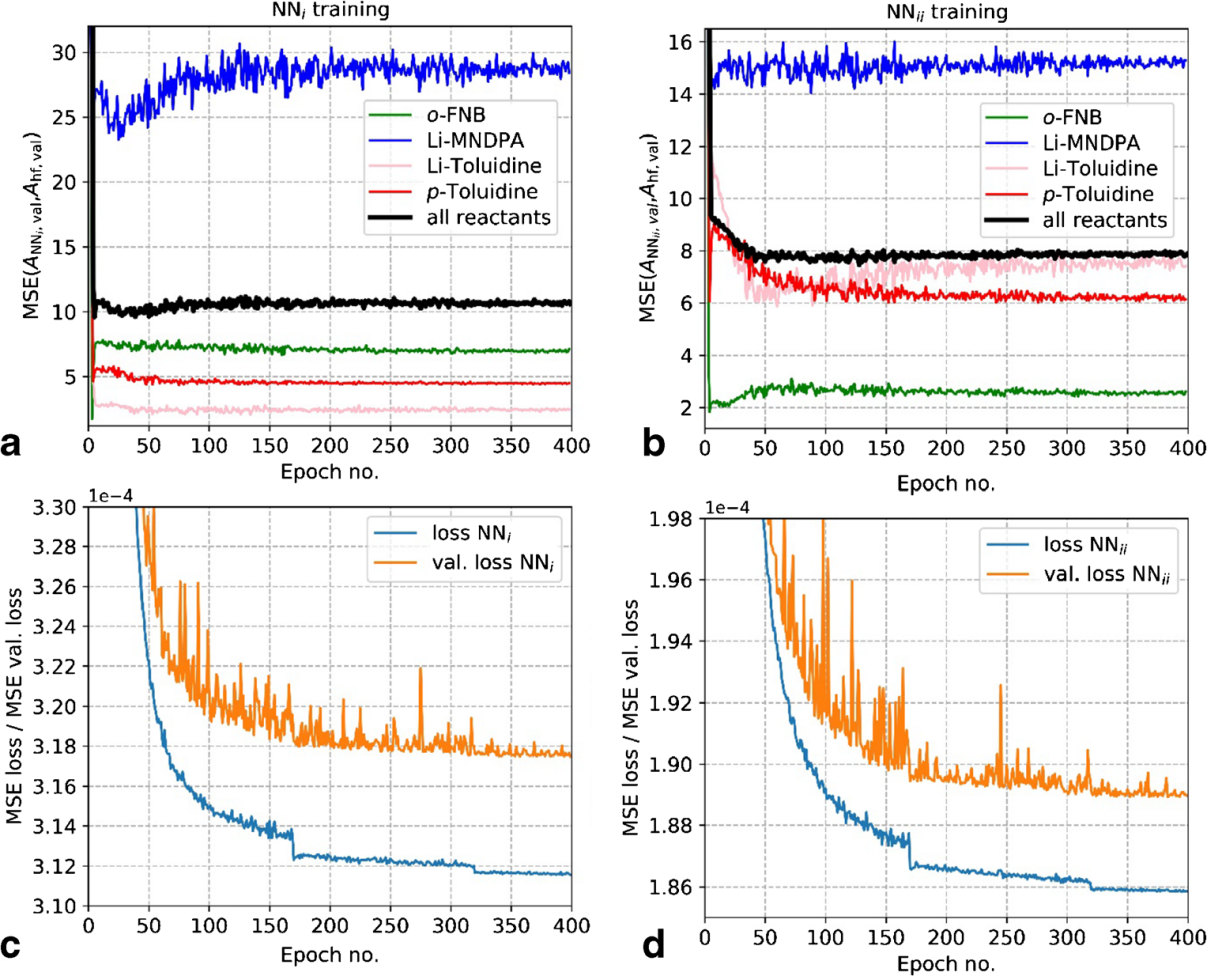
Training progress for all reactants expressed as the MSE (*A*_hf,val_, *A*_lf,ANN,val_) (**a**, **b**), and training/validation loss (**c**, **d**). The “pure component dataset” NN_*i*_ model results are shown in **a** and **c**, and the “spectral model dataset” NN_*ii*_ model results are shown in **b** and **d**

We follow the training and validation procedure from reference [29]. The training is conducted on synthetic training data, but along with the implicit validation on synthetic training data, additional validation is already conducted on experimental measurement data during the training after each training epoch (here, mean squared error deviation from *A*_hf_). With this evaluation step, the performance on the actual measurement data can be analyzed already during the training with the synthetic data. This significantly eases the selection of the hyperparameters of the model architecture and the parameters for the training. It also allows one to identify overfitting of the model to the training data (see Fig. 5). For this additional evaluation step, the measured low-field NMR spectral dataset (*S*_lf_) is separated into a validation dataset (*S*_lf,val_) and a test dataset (*S*_lf,test_). *S*_lf,val_ is used to predict *A*_lf,ANN,val_ during the training after each training epoch in order to quantify the model performance on actual measured spectra with the MSE (*A*_hf,val_, *A*_lf,ANN,val_) (cf. Fig. 5). The test dataset *S*_lf,test_ is finally used to quantify the prediction performance of the optimized ANN models by computing the mean squared error MSE (*A*_hf,test_, *A*_lf,ANN,test_).

Before the training, the ANN training data is normalized to the range [0,1]. The measured low-field spectra were respectively normalized to *S*_lf_′ = *S*_lf_/max (*X*_*i*/*ii*_) before prediction. The label range was also normalized to the range [0,1] by computing *A*_lf,synth_′ = *A*_lf,synth_/max (*A*_lf,synth_). The ANN predictions (*A*_lf,ANN_) are therefore back transformed by multiplication with the same factor max (*A*_lf,synth_) before the MSE analysis. The Adam optimizer (*β*_1_ = 0.9, *β*_2_ = 0.999) was used for all training sessions [30, 31]. The training and prediction were conducted on an Intel i7-8565U CPU (1.8 GHz), using Keras (v. 2.2.4) and TensorFlow (v. 1.15). The models are small enough to do this efficiently on a CPU.

#### ANN architecture, hyperparameter search, and training

The ANN model architecture and training parameters were selected using the above-described training procedure. Two different neural network types were considered: MLPs (a series of fully connected layers) and CNNs.

Initial coarse hyperparameter exploration showed that convolutional neural networks generally perform better than MLPs for this task. The MLP prediction accuracy only comes close to that of CNNs if multiple fully connected hidden layers (> 3) with a large number of nodes (descending with increasing layer number) are used. These models contain trainable parameters in the order of > 40 million, far more than CNN models. This is also not favorable since real-time prediction with limited hardware requirements is intended. The focus of the parameter search was therefore on CNN-based architectures. The following architectures and hyperparameters were tested with respect to minimizing MSE (*A*_lf,ANN,val_, *A*_hf,val_): type of convolutional layers (Conv1D vs. LocallyConnected1D), number of convolutional layers, number of convolutional layers before pooling layer, pooling layer type (AveragePooling1D, MaxPooling1D), size of pooling or strides, number of filters, kernel size, number of convolution/pooling blocks, type of activation function after convolutional layers (rectified linear unit (ReLU), exponential linear unit (ELU), hyperbolic tangent function tan *h*), number of dense layers after the convolutional blocks, number of nodes in dense layers, activation function after dense layers, activation function of output layer (linear, ReLU, ELU), learning rate, batch size, and amplitude of additive white Gaussian noise (AWGN).

The parameter testing was conducted with the ANN trained using the spectral model dataset (*X*_*ii*_) since its predictions were consistently more precise than training with the pure component spectral dataset (*X*_*i*_), and the relative performance did not deviate much between the two datasets at initial coarse parameter cross-testing. The final spectral model dataset ANN is labeled NN_*ii*_ and the final “pure component spectral analysis” is labeled NN_*i*_. The model NN_*i*_ is eventually trained with the parameters obtained from the parameter search using *X*_*ii*_.

From the hyperparameter analysis, it can be concluded that a very compact and shallow architecture can be used: a single convolutional layer with a kernel size of 9, strides of 9, with only 4 filters, and a single flatten layer connected to the four-node output layer with ReLU activation is sufficient. A single LocallyConnected1D convolutional layer with ELU activation showed improved performance compared to a Conv1D layer. Increasing the ANN depth, kernel size, and filter numbers or adding additional fully connected layers after the convolutional blocks did not improve the model’s prediction accuracy. The final model consists of only 10,532 trainable parameters and can, therefore, also be integrated into embedded systems with typically limited memory and computation power [32]. Using mean squared error loss function, ADAM optimizer, and additive white Gaussian noise with zero mean and a standard deviation of 0.04 on *X*_*i*/*ii*_ showed the best results. To train the final models, the learning rate was reduced in steps from 10^−4^ to 3 · 10^−5^ to 10^−5^ at epochs 170, 240, and 310, respectively. A batch size of 1024 was used during training.

The prediction performance of the NN_*i*_ model (trained with *X*_*i*_) and the NN_*ii*_ model (trained with *X*_*ii*_) with respect to the measured low-field validation data MSE (*A*_hf,val_, *A*_lf,ANN,val_) as well as loss and validation loss on the synthesized training *X*_*i*/*ii*_ data for all reactants and the individual reactants during the training are shown in Fig. 5.

It has to be noted that the ANNs have been trained to predict over the entire combination of component area ranging from 0 to 180 for each of the four reactants. However, we were only able to test the performance on a relatively limited concentration range and a combination of reactant concentrations. The MSE on measured validation data exhibits a minimum after < 100 epochs (see Fig. 5a, b), increases with further training, and converges after about 200 epochs while loss and validation loss on synthetic training data exhibit a continuous exponential decrease.

Although a better performance on the validation data and also on the test data that is available to us can be achieved after 50 epochs, we continued the training of the final model to 400 epochs since we intended to train a model that can be applied reliably to the entire concentration range of all reactants. Stopping the training at a minimum of the MSE (*A*_hf,val_, *A*_lf,ANN,val_) would most likely result in a considerably reduced precision for a wide range of reactant concentration combinations, which are not well represented by the measured validation dataset. Due to the limited validation and test dataset, we cannot test the model’s convergence for the entire reactant concentration range and decided to continue training until the convergence of the MSE (*A*_hf,val_, *A*_lf,ANN,val_).

We conduct the evaluation on the measured test dataset with a model that has been trained for 400 epochs and converged with respect to MSE (*A*_hf,val_, *A*_lf,ANN,val_). The ANN prediction performance on the test dataset (*S*_lf,test_) is quantified and compared to the IHM analysis in the following section.

## Results and discussion

**Fig. 6:**
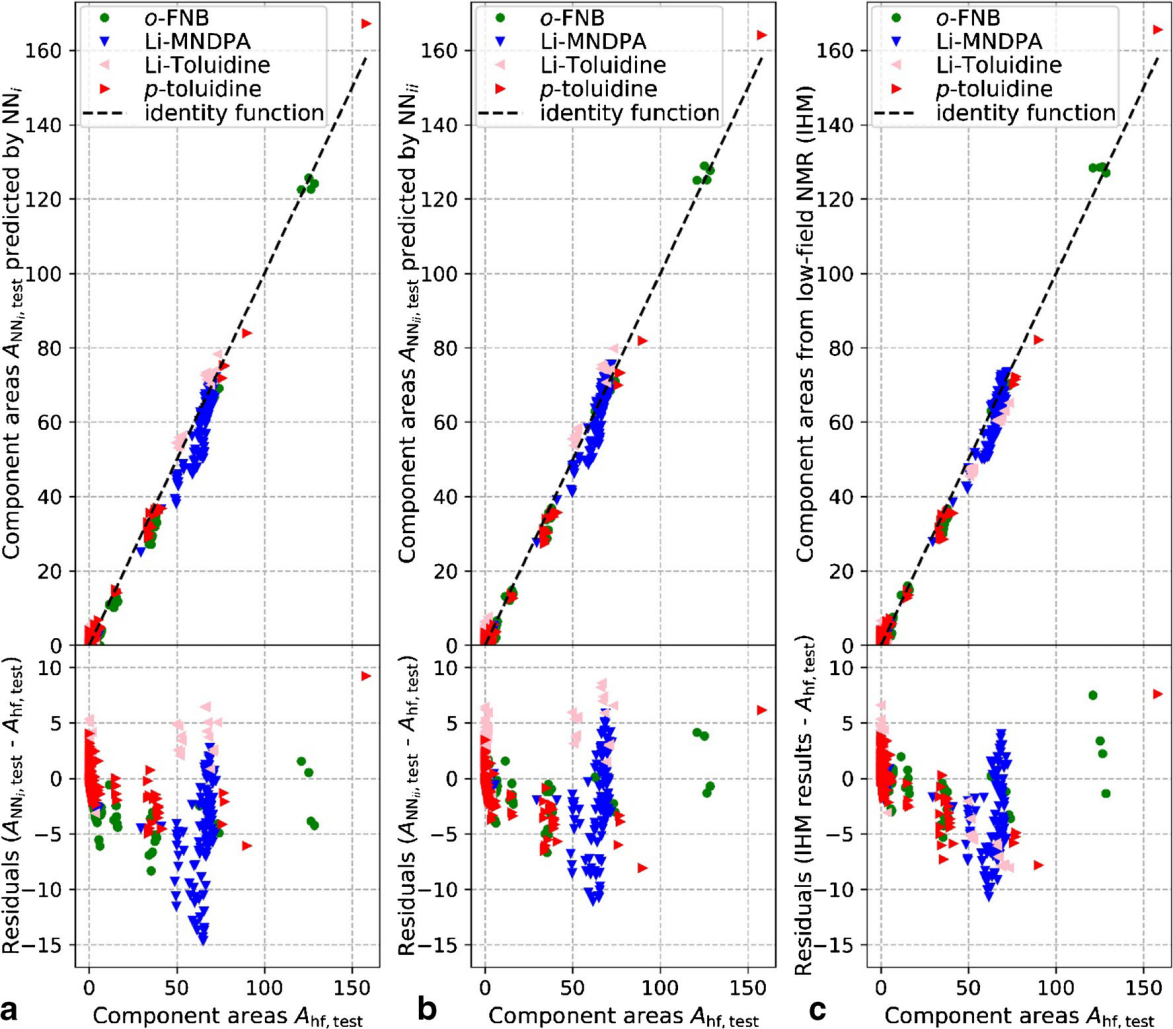
Parity plots of component areas (*A*_NN,test_) relative to the high-field NMR results (*A*_hf,test_) computed from low-field NMR input (*S*_lf,test_) (top) along with their residuals (*A*_NN,test_ − *A*_hf,test_) (bottom). **a** Predicted by NN_*i*_ (trained with “pure component spectral dataset”). **b** Predicted by NN_*ii*_ (trained with “spectral model dataset”). **c** IHM result

The prediction performance of the two neural networks that were trained with the pure component spectral dataset (NN_*i*_) and with the spectral model dataset (NN_*ii*_) is compared to the high-field NMR results (*A*_hf_). Both networks were trained with the same architecture using the parameters summarized above. The parity plots of the NN_*i*_ predictions and the NN_*ii*_ predictions as well as the parity plot of the IHM evaluation results from the test dataset (*S*_lf,test_) are shown in Fig. 6.

**Fig. 7:**
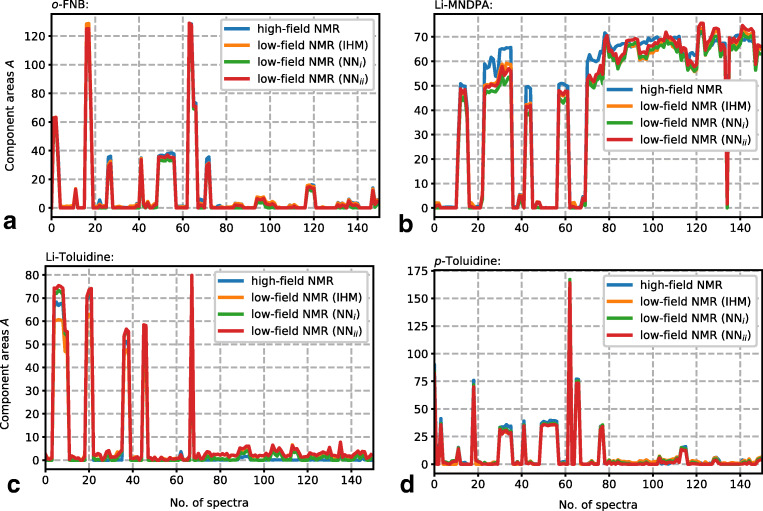
Component areas (*A*) predicted from low-field NMR data along with experimental and high-field NMR spectra as reference data for **a***o*-FNB, **b** Li-MNDPA, **c** Li-toluidine, and **d** toluidine

The entire reactant concentration results (*A*_lf,test_) computed from *S*_lf,test_ by NN_*i*_, NN_*ii*_, and IHM as well as the high-field NMR results are shown in Fig. 7.

A comparison of the mean squared errors of the single reactants is summarized in Table 3. The NN_*ii*_ results (trained with spectral model dataset) overall exhibit the smallest error, slightly lower than the IHM results. NN_*ii*_ results also exhibit a high MSE similarity to the IHM results for the single reactants. The NN_*i*_ results (trained with pure component spectral dataset) exhibit the highest overall deviation from *A*_hf,test_ but improved MSE for two of the reactants (Li-toluidine and toluidine). Training with a mixed dataset (pure component spectral dataset and spectral model dataset with a 50:50 split) did not lead to an overall improved MSE (*A*_hf,test_, *A*_lf,test_).

**Table 3:** Comparison of mean squared error MSE (*A*_hf,test_, *A*_lf,test_) of computed reactant concentrations (as signal areas) from low-field NMR test data (*S*_lf,test_) using neural network predictions (ANN_*i*_ and ANN_*ii*_) and IHM analysis in comparison to high-field NMR results

Reactant	NN_*i*_	NN_*ii*_	IHM
*o*-FNB	6.37	2.28	*2.17*
Li-MNDPA	29.16	*16.04*	17.99
Li-toluidine	*3.35*	8.80	8.45
Toluidine	*3.44*	4.55	4.79
All reactants	10.58	*7.92*	8.35

The lowest MSE for each reactant is styled in italics

The prediction time of the ANNs is about 33 μs/spectrum for 1000-spectrum batch and about 0.9 ms for a single-spectrum batch (on Intel i7-8565U CPU (1.8 GHz), Keras (v. 2.2.4), TensorFlow (v. 1.15)). The computation time for IHM predictions is in the order of several seconds and depends on a nonlinear optimization with a termination criterion.

## Conclusion

In order to validate a machine-supported method to build a calibration model, reliable and meaningful low-field NMR spectroscopy data of an exemplary reaction synthesis (300 spectra) was processed and compared with that of high-field NMR spectroscopy as reference method. Normally, small experimental datasets are not diverse enough to apply machine-supported methods. The experimental data was therefore artificially extended, applying a new method which introduced additional variance by a systematic variation of peak shape and position in the spectral model based on physical principles. This allowed to increase the size of the small initial dataset to the amount of 300,000.

In order to better understand how this procedure works, two variants to produce the synthetic spectra were employed. In dataset (*i*), the pure substance data was shifted only on the spectral frequency axis, and in dataset (*ii*), a more complex method was applied, where size, shape, and spectral position of the pure substance data were separately varied randomly. In both cases, the amplified synthetic spectral datasets were successfully used to train ANNs.

In the past, these types of measurements could only be evaluated by physical methods, such as the direct integration of spectral bands (e.g., according to the principle of Lambert Beer’s law) or based on physical models (e.g., as in the case of IHM). This makes the procedure time intensive and expensive. However, the ANN method provides values comparable to those of IHM. The ANN method is advantageous because it requires very little computing power for predictions and is orders of magnitudes faster than IHM. This is advantageous if the prediction is to be implemented in embedded systems such as “smart sensors.” Another advantage of the ANN method is that it does not rely on discrete decisions of a nonlinear optimization scheme, which is used during the spectral model fitting within the IHM workflow. These discrete decisions tend to yield biased or “jumping” predictions, e.g., when fitting small peaks and overlapping larger peaks and such effects are indirectly caused by penalty functions within the optimization algorithm, e.g., when a peak is surpassing a given threshold. Synthetic spectra can therefore also be used for the evaluation of other data-driven methods of data analysis, such as PLS-R or support vector machines.

A certain limitation of the ANN methodology is that the resulting model may only reproduce those changes that are within the training label space. Consequently, the application to ranges outside the training dataset will be limited. If the variance within the prediction spectra is not or not completely covered by the spectral model or the synthetic spectra, the ANN methodology will therefore not be robust. This applies specially to baseline distortions, the occurrence of unexpected components, or disturbances in the spectrum or similar. In our case, the variance of the prediction spectra was based on previous knowledge and experience. As it is the case for many specific machine learning applications, specific domain knowledge is a key to achieve high-performance predictions also for our application. Further research is needed on how this newly developed method can be systematically incorporated into the design of the model. However, some application scenarios are already feasible. Firstly, in the case of an addition of an analyte, the training dataset can be regenerated within a short time (seconds to minutes) in order to train an extended neural network for the prediction of the concentrations in the new reaction mixture. This would be possible within minutes on a standard PC. The extended (fast) ANN models can then be reloaded into the embedded systems as model updates. Secondly, the procedure described above for multiplying the datasets could also have an impact on the use of historical data and make it usable in retrospect.

## Electronic supplementary material

ESM 1 (PDF 283 kb)

## Data Availability

The data used for training and validation of the ANNs are available in a public data repository (10.5281/zenodo.3677139).

## Abbreviations

*A*_hf_: Measured high-field NMR areas (reference)
*A*_hf,test_: Measured high-field NMR areas (reference) of test dataset
*A*_hf,val_: Measured high-field NMR areas (reference) of validation dataset
*A*_lf,ANN_: Predicted areas of ANN model
*A*_lf,ANN,val_: Predicted areas of ANN model for validation dataset (*S*_lf,val_)
*A*_lf,IHM_: Predicted areas of IHM
*A*_lf,synth_: Areas of pure components of synthetic mixture spectra
*k*: Index for component model
*K*: Total number of pure component models
NN_*i*/*ii*_: Final artificial neural network trained with *X*_*i*/*ii*_
*S*_hf_: Experimental high-field NMR spectra
*S*_lf_: Experimental low-field NMR spectra
*S*_lf,val_: Experimental low-field NMR spectra used for validation
*S*_lf,test_: Experimental low-field NMR spectra used for testing
*w*: Component weight
*X*_*i*_: Dataset based on combinations of measured pure component spectra
*X*_*ii*_: Dataset based on a spectral model
*X*_*i*/*ii*,train_: Training dataset of synthetic data
*X*_*i*/*ii*,val_: Validation dataset of synthetic data
*α*: Peak maximum
*β*: Gauss–Lorentz weight
*β*_1_: Exponential decay rate first moment estimates, Adam optimizer
*β*_2_: Exponential decay rate second-moment estimates, Adam optimizer
*γ*: Peak width
*θ*_GS_: Group shift parameter
*θ*_P_: Peak parameters of the pure component model
*θ*_PB_: Peak broadening parameter
*θ*_S_: Component shift parameter
*ν*: Spectral axis (in ppm)
*χ*: Spectral mixture model
*ω*: Peak position
