# Supplementary material for: Artificial neural networks for quantitative online NMR spectroscopy
Source: Anal Bioanal Chem. 2020 May 9;412(18):4447–59. doi: 10.1007/s00216-020-02687-5 (PMC7320049; doi:10.1007/s00216-020-02687-5)
Supplement: Supplementary file 1 — (PDF 283 kb) [file 216_2020_2687_MOESM1_ESM.pdf]

Analytical and Bioanalytical Chemistry

Electronic Supplementary Material

Artificial neural networks for quantitative online NMR spectroscopy

Simon Kern, Sascha Liehr, Lukas Wander, Martin Bornemann-Pfeiffer, Simon Müller, Michael Maiwald, Stefan Kowarik

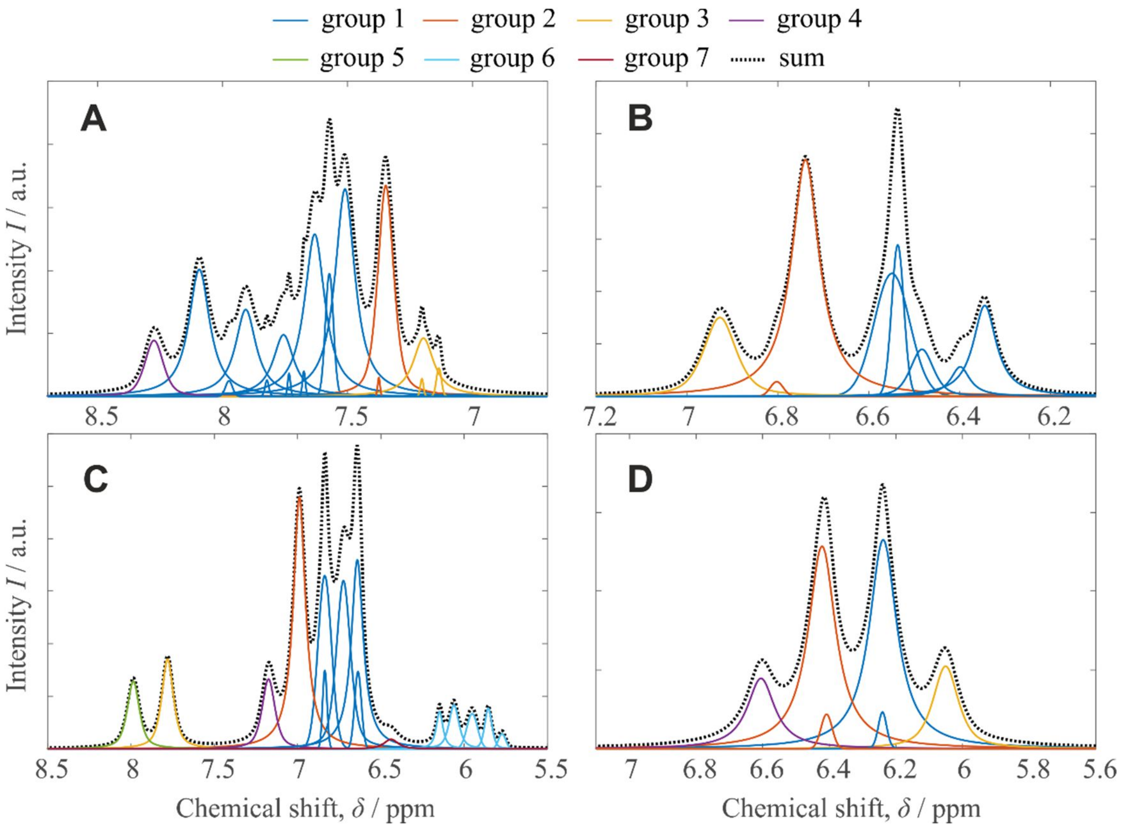

Fig. S1 Visualization of the peak groups for the pure component models which were created in PEAXACT 4 and grouped automatically using MATLAB. A: *o*-FNB; B: Li-toluidine; C: Li-MNDPA; and D: toluidine.

For further information see also Ref. [5] and [8]:

[5] Kern S, Wander L, Meyer K, Guhl S, Mukkula ARG, Holtkamp M, et al. Flexible automation with compact NMR spectroscopy for continuous production of pharmaceuticals. Anal Bioanal Chem. 2019; 411: 3037–3046.

[8] Kern S, Meyer K, Guhl S, Gräßer P, Paul A, King R, et al. Online low-field NMR spectroscopy for process control of an industrial lithiation reaction—automated data analysis. Anal Bioanal Chem. 2018; 410: 3349–3360.
